# Supplementary material for: The interactome of intact mitochondria by cross-linking mass spectrometry provides evidence for coexisting respiratory supercomplexes
Source: Mol Cell Proteomics. 2017 Dec 8;17(2):216–32. doi: 10.1074/mcp.RA117.000470 (PMC5795388; doi:10.1074/mcp.RA117.000470)
Supplement: Supplemental Data [file supp_RA117.000470_133922_0_supp_23978_fzffwf.zip › spectra_annotation/mito_spectra_annotation/28-1-17-1-13-1.pdf]

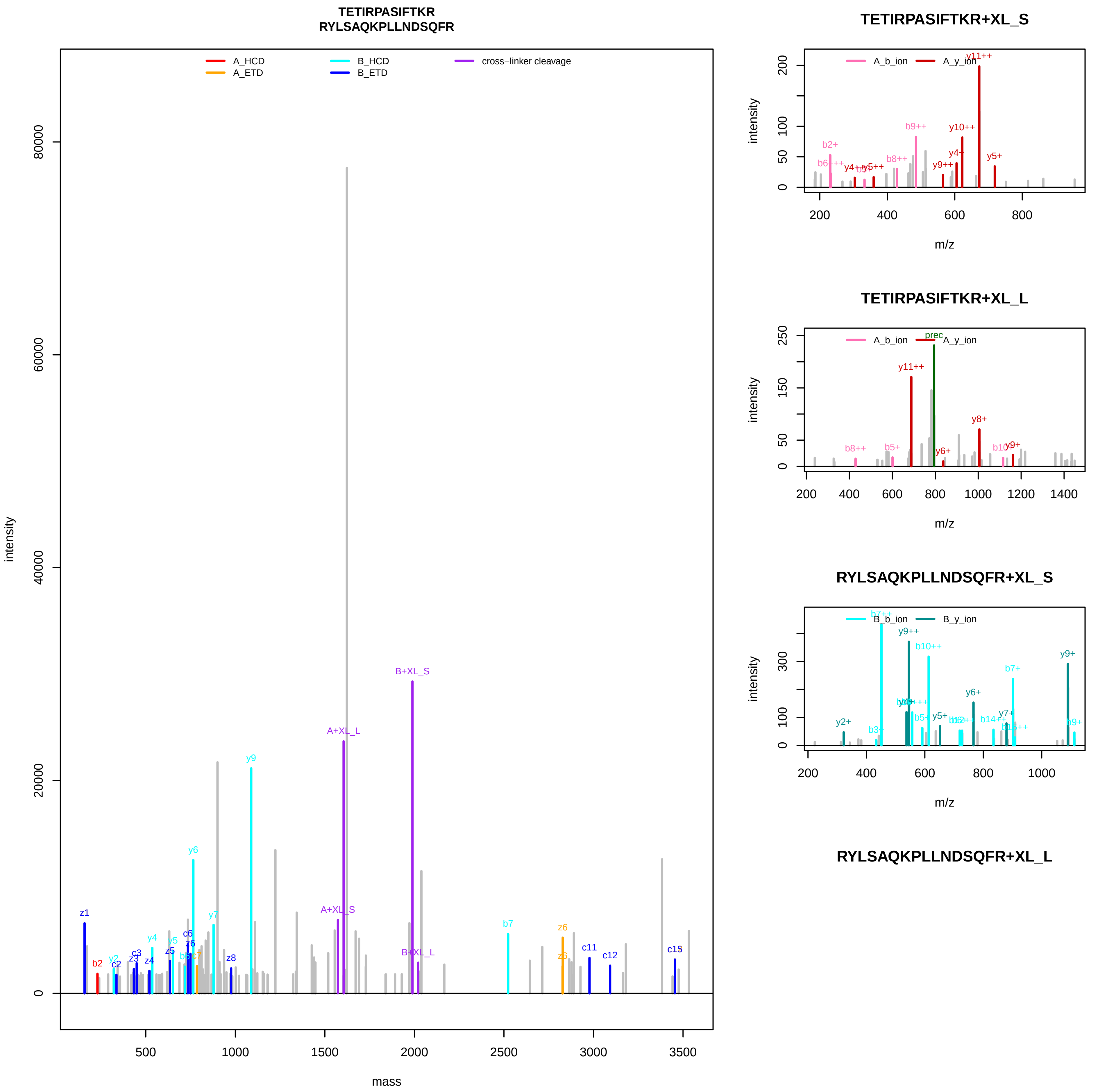
TETIRPASIFTKR
RYLSAQKPLLNDSQFR
A_HCD
A_ETD
B_HCD
B_ETD
cross−linker cleavage
intensity
mass
0
20000
40000
60000
80000
500
1000
1500
2000
2500
3000
3500
z1
b2
y2
c2
z3
c3
z4
y4
z5
y5
c6
z6
y6
y7
z8
y9
A+XL_S
A+XL_L
B+XL_S
B+XL_L
b7
z6
c7
c11
c12
c15
TETIRPASIFTKR+XL_S
A_b_ion
A_y_ion
y11++
b2+
b9++
y10++
b8++
y4+
y5+
y9++
y4++
y5++
m/z
TETIRPASIFTKR+XL_L
prec
b8++
b5+
y6+
y8+
y9+
RYLSAQKPLLNDSQFR+XL_S
B_b_ion
B_y_ion
b7++
y9++
b10++
y2+
b3+
b5+
y5+
y6+
b7+
y7+
b14++
y9+
b9+
RYLSAQKPLLNDSQFR+XL_L
